# Fall-related functional impairments in patients with neurological gait disorder

**DOI:** 10.1038/s41598-020-77973-4

**Published:** 2020-12-03

**Authors:** Angela Ehrhardt, Pascal Hostettler, Lucas Widmer, Katja Reuter, Jens Alexander Petersen, Dominik Straumann, Linard Filli

**Affiliations:** 1 Department of Neurology, University Hospital and University of Zurich, Zurich, Switzerland; 2 Spinal Cord Injury Center, Balgrist University Hospital, Zurich, Switzerland; 3 Swiss Center for Clinical Movement Analysis (SCMA), Balgrist Campus AG, Zurich, Switzerland

**Keywords:** Neuroscience, Motor control, Neurological disorders

## Abstract

Falls are common in patients with neurological disorders and are a primary cause of injuries. Nonetheless, fall-associated gait characteristics are poorly understood in these patients. Objective, quantitative gait analysis is an important tool to identify the principal fall-related motor characteristics and to advance fall prevention in patients with neurological disorders. Fall incidence was assessed in 60 subjects with different neurological disorders. Patients underwent a comprehensive set of functional assessments including instrumented gait analysis, computerized postural assessments and clinical walking tests. Determinants of falls were assessed by binary logistic regression analysis and receiver operator characteristics (ROC). The best single determinant of fallers was a step length reduction at slow walking speed reaching an accuracy of 67.2% (ROC AUC: 0.669; p = 0.027). The combination of 4 spatio-temporal gait parameters including step length and parameters of variability and asymmetry were able to classify fallers and non-fallers with an accuracy of 81.0% (ROC AUC: 0.882; p < 0.001). These findings suggest significant differences in specific spatio-temporal gait parameters between fallers and non-fallers among neurological patients. Fall-related impairments were mainly identified for spatio-temporal gait characteristics, suggesting that instrumented, objective gait analysis is an important tool to estimate patients' fall risk. Our results highlight pivotal fall-related walking deficits that might be targeted by future rehabilitative interventions that aim at attenuating falls.

## Introduction

Falls are common in elderly people and are often associated with serious injuries, reduced mobility and loss of independency^1^. 25–35% of people older than 65 years fall regularly and more than half of falls in these patients occur during walking^2^. There is a multitude of studies investigating functional determinants or predictors of falls in healthy elderly subjects. Identified functional impairments that differentiate healthy fallers from non-fallers are heterogeneous and depend on the functional outcomes that are assessed in the particular studies^3^. Most common techniques assessing fall risk comprise motor performance tests, questionnaires, and laboratory-based, instrumented measurements (e.g. force platforms, computerized walkways, accelerometers etc.). Spatio-temporal gait parameters, in particular reduced step length and increased variability of stance and stride time, were shown to be valid determinants of falls in elderly subjects^2–13^. In addition, computerized outcomes of postural stability (e.g. sway assessment) and clinical walking outcomes (e.g. timed-up and go (TUG), functional gait assessment (FGA)) revealed significant correlations with fall risk in elderly people^14–17^.

In contrast to healthy subjects, potential predictors of falls are poorly investigated in patients with neurological disorders. Fall incidence is reported 2–4 times higher in patients with neurological disorders than in healthy subjects of similar age^18,19^ and 46% of the neurological patients reveal one or more falls per year^20^. There are studies examining fall-associated functional impairments regarding clinical assessments^21,22^, postural outcomes^23,24^ and computerized gait analysis^25–28^. There is, however, only sparse knowledge on a valid, standardized determinant of falls that might apply to different neurological gait pathologies. Moreover, there are virtually no studies performing a comprehensive functional test battery including manifold biomechanical parameters of stance and gait, as well as of standardized clinical walking tests to screen for predominant functional impairments that are characteristic of fallers with neurological movement disorders.

The aim of this study was to identify functional impairments regarding posture and gait that are most characteristic of fallers in patients with neurological gait disorders. In contrast to earlier studies, we examined determinants of falls in a heterogeneous group of neurological patients comprising, among others, patients with multiple sclerosis, polyneuropathy, vertigo syndromes, cerebrovascular diseases and idiopathic normal pressure hydrocephalus. Given the heterogeneous literature on determinants of falls, we screened a large, multimodal set of different postural and clinical outcome measures as well as gait parameters at different walking speeds for their ability to differentiate fallers from non-fallers. Gait assessments at differential walking speeds were performed to place varying demands on locomotor function (e.g. slow walking speeds demand for high dynamic stability, faster speeds require enhanced force and coordinative abilities^29^). Our findings might help to improve upon the understanding of falls in neurological patients and thereby promote the designing of therapeutic strategies that aim at preventing or reducing the incidence of falls in neurological patients.

## Methods

### Participants

**Figure 1:**
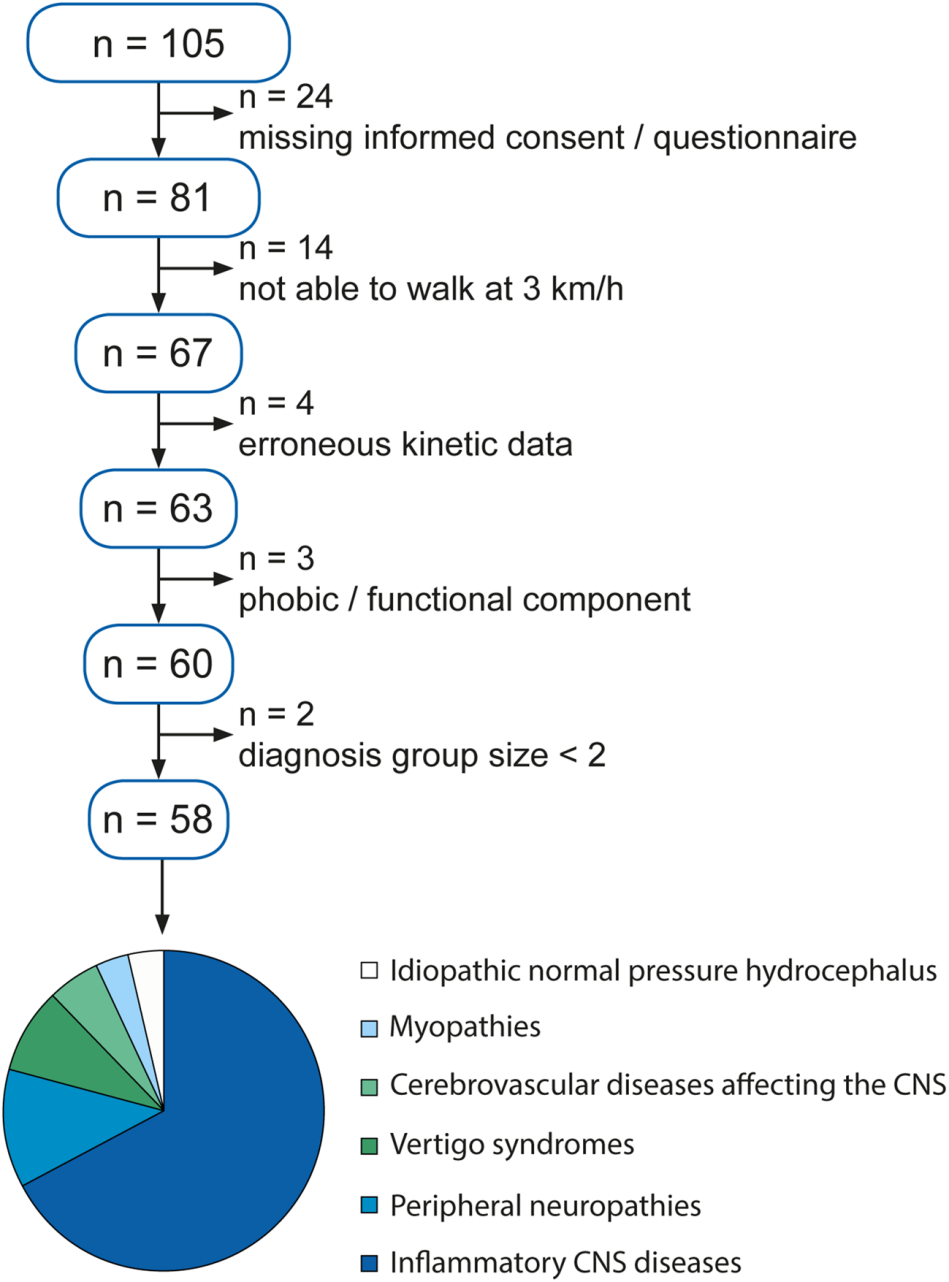
Flowchart of the sampling procedure.

Functional data was retrospectively analyzed from participants that were routinely examined at the Locomotion Research Laboratory of the Department of Neurology at the University Hospital Zurich. There was no specific recruitment of patients for this study. All participants provided written informed consent regarding a further use of their clinical data for the purpose of clinical research. All measurements were performed in accordance with the Declaration of Helsinki. The procedures were approved by the Zurich cantonal ethics committee (project ID: 2017-01459). Patients with a defined neurological diagnosis and no major orthopedic, cardiovascular or pulmonary disorders affecting walking function were included (Tables 1, 2). For inclusion, patients needed to be able to complete all clinical walking tests, the postural assessments and the kinetic walking trials. All patients were able to understand the content of the fall questionnaire. Participants with suspected psychogenic gait disorders or major cognitive deficits were excluded from the analysis. Patients who were not able to walk without holding onto the handrails of the treadmill at 1, 2 and 3 km/h where excluded: Furthermore, we excluded groups with only one participant (Fig. 1, Table 1).

**Table 1:** In- and exclusion criteria.

Inclusion criteria	Exclusion criteria
• Signed informed consent • Completed fall questionnaire • Complete dataset of clinical walking tests (timed 25-foot walk, 6-min walk test, timed up and go, functional gait assessment) • Complete set of postural assessments (body sway during normal standing, Romberg position with eyes open, Romberg position with eyes closed) • Complete dataset of treadmill walking without handrail support at 1,2 and 3 km/h • Patients ≥ 18 years with a defined neurological diagnosis	• Lack of informed consent or fall questionnaire • Incomplete dataset of clinical walking tests, postural and • locomotor assessments • Patients that were not able to understand the content of the fall questionnaire • Participants with suspected psychogenic gait disorders or major cognitive deficits • Major orthopedic, cardiovascular or pulmonary disorders affecting walking function • Patients with a secondary neurological diagnosis

**Table 2:** Demographic and clinical characteristics of the study population. *CNS* central nervous system, *SD* standard deviation, *MS* multiple sclerosis, *CIS* clinically isolated syndrome.

	Patients
Number of patients	58
Age (years), mean ± SD	52.5 ± 14.5
Gender, proportion of female	25/58
Fallers, proportion of fallers	29/58
**Neurological condition, number of patients**	
Inflammatory CNS diseases (MS: n = 37; CIS: n = 2) Peripheral neuropathies Vertigo syndromes Cerebrovascular CNS diseases Myopathies Idiopathic normal pressure hydrocephalus	39 7 5 3 2 2

## Experimental procedures

Participants were asked to complete a questionnaire assessing the incidence of falls within 6 months prior to data analysis. Considering the potential for recall bias the period of retrospective fall assessment was limited to six months. A fall was defined as an unexpected event where a patient lost his balance and reached a position on the floor^9,11,18^. Collapses because of internistic events such as seizures, ischemic attacks or syncopes were not defined as falls. Participants who had at least one fall within the last 6 months where classified as “fallers”.

All participants underwent a comprehensive gait analysis and performed different posturographic measurements. Clinical gait tests consisted of the timed 25-foot walk (T25FW)^30^ assessing maximal walking speed, the 6-min walk test (6MWT)^31^ measuring short-time endurance, the timed up and go test (TUG)^32^ assessing functional mobility and daily locomotor function as well as the functional gait assessment (FGA)^33^ measuring dynamic stability and balance.

Instrumented, treadmill-based gait analysis was used to objectively assess patients’ walking patterns (i.e. quality of walking) at 1, 2 and 3 km/h. Different gait speeds were used to assess distinct sensorimotor abilities (e.g. high stability requirements at slow speed vs. enhanced force and coordinative requirements at faster speeds). Comprehensive gait profiles consisting of 20 kinetic gait parameters were produced for each patient at each gait velocity. These walking parameters served to assess different aspects of locomotion such as step length, gait phases, dynamic instability, asymmetry and gait variability, thus being able to comprehensively characterize patients’ walking pathologies (Table 3). All participants walked barefoot for at least 30 s per trial on an instrumented treadmill (120 Hz, FDM-T, Zebris Medical GmbH, Germany) without holding onto the handrails. Prior to data recording, all participants were familiarized to treadmill walking for at least 7 min at each gait speed (1, 2 and 3 km/h) to prevent major adaptations of the gait pattern and to reduce the fear of falling^34^. After this initial familiarization period, kinetic gait data was recorded over 30 s at each velocity (1, 2 and 3 km/h). Participants were instructed not to talk or gesticulate and to naturally move/swing their arms. Moreover, patients were asked to fix their gaze onto a cross projected on a screen (22″ LCD monitor) positioned at eye height in front of the treadmill at any time during the recordings^35^. After the training and between the trials patients were allowed to have a short break if required.

**Table 3:** Gait and postural assessments performed in this study. Walking parameters indicative of various locomotor aspects (e.g. stability, asymmetry, variability) were assessed at gait speeds of 1 km/h, 2 km/h and 3 km/h (subtable on the top).

Spatio-temporal kinetic gait parameters		
Locomotor domain	Gait parameters	Units
Limb excursion	Step length (left, right)	mm
	Step time (left, right)	ms
Gait phases	Stance phase (left/right)	%
	Swing phase (left/right)	%
	Double-limb support	%
Stability	Step width	mm
Asymmetry	Step length asymmetry	%
	Step time asymmetry	%
	Swing phase asymmetry	%
Variability	COV step length (left, right)	%
	COV step time (left, right)	%
	COV step width	%
	CoP variability ant-post	mm
	CoP variability med-lat	mm

Clinical walking tests		
Test	Readouts	Units
Timed 25-foot walk (T25FW)	Maximal walking speed	s
6-min walk test (6MWT)	Short-term endurance	m
Timed-up and go (TUG)	Mobility, daily walking function	s
Functional gait assessment (FGA)	Dynamic stability, balance	Points

Postural stability measures		
Test	Stability measures	Units
Normal stance (feet 20cm apart)	Sway velocity	mm/s
	Sway area (95% confidence)	mm^2^
Romberg stance (eyes open)	Sway velocity	mm/s
	Sway area (95% confidence)	mm^2^
Romberg stance (eyes closed)	Sway velocity	mm/s
	Sway area (95% confidence)	mm^2^

Four clinical walking tests were assessed to quantify patients’ walking performance (intermediate table). Three different postural test conditions were performed to measure patients’ postural stability (table at the bottom). *CoP* center of pressure, *COV* coefficient of variance, *mm* millimeter, *min* minute, *ms* milliseconds.

Postural stability was quantified by center of pressure (CoP) sway measurements during normal standing (20 cm distance between left and right hallux) and during the Romberg test (i.e. feet close together) with eyes open and closed^36^. Postural assessments were performed on the instrumented treadmill. Participants performed four trials of 15 s for each postural condition. CoP sway velocity (mm/s) and the 95% confidence interval of the ellipse sway area (mm^2^) were used as outcome parameters of postural stability. For each test condition, the trial with the lowest sway velocity (i.e. the most stable trial) was selected for analysis.

Kinetic data of the gait and posture trials were acquired by a pressure plate that was integrated in the treadmill (DMTHM-M-2i System, Zebris Medical GmbH, Germany). Parameter raw data per step cycle and patient was extracted from the Zebris FDM software and further processed in Maltab using customized scripts (Matlab 2017b, Mathworks Inc., Natick, USA).

### Data analysis

Statistical analysis was performed with SPSS statistical software (V23.0; IBM Corp., Armonk, NY). Group differences in kinetic gait patterns, clinical walking performance and postural stability between fallers and non-fallers were assessed by 2-way ANOVA with the factors fallers and parameters. Post-hoc corrections for multiple testing (Sidak’s correction) was performed to assess group differences on the level of single parameters. Binary logistic regression (Wald forward stepwise) was used to identify determinants of falls and to determine their contribution to separate fallers from non-fallers^13^. Results of the kinetic gait analysis, the clinical gait tests and the posturographic assessments (i.e. 70 factors) were included in the binary logistic regression. The dependent variable was defined as fall status, whereby fallers were defined as patients experiencing at least one fall within the last 6 months prior to the clinical examination. Stepwise forward regressions were set at p > 0.05 for entry and removal of a variable. All analyses have been adjusted for the covariates age and gender.

Fall-related functional parameters as defined by binary logistic regression analysis were further assessed by receiver operator characteristics (ROC) with bootstrapping to obtain 95% confidence intervals. ROC analysis was performed for the strongest single determinant of falls, as well as for the combinations of fall determinants.

## Results

Fifty-eight patients (25 females, age: 52.5 ± 14.5 years) with gait disorders due to different neurological conditions were analyzed (Table 2). Five patients with suspected psychogenic origin of gait disorders were excluded from the analysis. Twenty-nine of 58 patients reported falls within the last 6 months prior to the functional assessments. None of the patients used assistive walking aids during the clinical walking tests or the instrumented gait analysis.

### Characterizing gait and postural deviations in fallers vs. non-fallers

**Figure 2:**
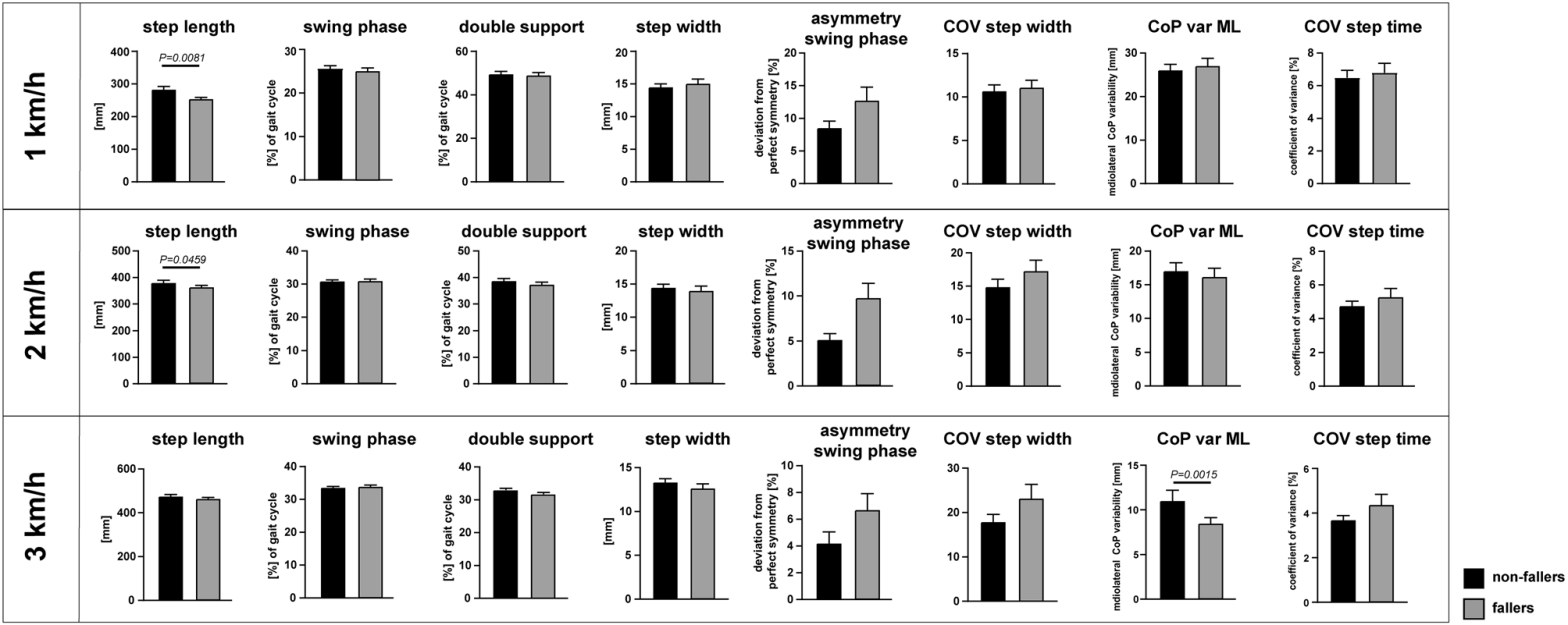
Key spatio-temporal gait parameters at different walking speeds in fallers vs. non-fallers. Spatio-temporal gait parameters were compared between fallers vs. non-fallers of our cohort. Significant differences (indicated by P-values above parameters) are based on 2-way ANOVA followed by Sidak's post hoc correlation for all 20 walking parameters at the respective gait speeds of 1 km/h, 2 km/h and 3 km/h. *CoP* center of pressure, *COV* coefficient of variance, *var* *ML* variability in mediolateral direction.

In a first step, we analyzed group differences in fallers vs. non-fallers regarding kinetic gait patterns, clinical walking performance and postural stability. There were no significant differences between fallers vs. non-fallers regarding parameters of postural stability (2-way ANOVA, factors “fallers x parameters”; P = 0.2611) and clinical walking performance (2-way ANOVA, P = 0.1369). Spatio-temporal gait parameters over all three assessed gait velocities (1, 2 and 3 km/h) also revealed no significant group differences (2-way ANOVA, P = 0.1715). Interestingly, the analysis of spatio-temporal gait parameters at individual gait velocities showed no group differences at slow walking speed (i.e. 1 km/h; 2-way ANOVA; P = 0.7580), but enhanced differences at higher gait velocities (2 km/h; P = 0.1726) which reached statistical significance at 3 km/h (2-way ANOVA, factor fallers; P = 0.0178; Fig. 2). At 1 and 2 km/h, reduction of step length was the only significant difference in fallers vs. non-fallers surviving the correction for multiple comparisons (P = 0.0081 and P = 0.0459 respectively; Sidak’s correction). At 3 km/h, mediolateral variability of the center of pressure was significantly reduced in fallers vs. non-fallers (P = 0.0015; Fig. 2).

### Determinants of fallers vs. non-fallers

After characterizing gait and postural differences in fallers vs. non-fallers, we screened for the most potent determinants of falls. A total of 70 independent variables (Table 3; 20 gait parameters at 3 different gait velocities, 6 posturographic parameters, 4 clinical walking tests) were analyzed by a binary logistic regression analysis with the dependent variable defined as faller status (Table 4). The covariates age (P = 0.417) and gender (P = 0.185) did not have an influence on the analysis. The best single variable discriminating fallers vs. non-fallers was step length (right leg) at 1 km/h reaching an accuracy of 67.2% (Nagelkerke coefficient of determination R^2^ = 0.111; specificity: 55.2%; sensitivity: 79.3%). Combining the three gait parameters (1) step length at 1 km/h, (2) CoP variability mediolateral at 3 km/h and (3) asymmetry of swing phase at 2 km/h resulted in an accuracy of 72.4% (Nagelkerke coefficient of determination R^2^ = 0.486; specificity: 72.4%; sensitivity: 72.4%) to classify fallers and non-fallers. The best accuracy in discriminating fallers vs. non-fallers (accuracy: 81.0%; specificity: 79.3%; sensitivity: 82.8%; Nagelkerke coefficient of determination R^2^ = 0.558) was achieved using the combination of four kinetic factors: (1) step length at 1 km/h, (2) CoP variability mediolateral at 3 km/h, (3) asymmetry of swing phase at 2 km/h and (4) COV (coefficient of variance) of step width at 3 km/h. The model using one determinant only (model A: step length at 1 km/h) revealed the highest sensitivity (i.e. correctly assigning fallers), however, the lowest specificity (i.e. correctly assigning non-fallers) of all logistic regression models. Posturographic parameters or clinical walking outcomes were not sufficiently powerful to improve upon the classification of fallers and non-fallers in our population.

**Table 4:** Prediction of fallers vs. non-fallers.

Observed	Predicted		
	Non-fallers	Fallers	Percentage correct
**A: Model comprises step length at 1 km/h**			
Non-fallers	17	13	56.7
Fallers	5	25	83.3
			**70.0**
**B: Model comprises A & CoP variability med-lat at 3 km/h**			
Non-fallers	20	10	66.7
Fallers	8	22	73.3
			**70.0**
**C: Model comprises B & asymmetry swing phase at 2 km/h**			
Non-fallers	22	8	73.3
Fallers	8	22	73.3
			**73.3**
**D: Model comprises C & COV step width at 3 km/h**			
Non-fallers	24	6	80.0
Fallers	5	25	83.3
			**81.7**

Classification tables illustrating observed and predicted outcomes of the binary logistic regression model using (A) the single most predictive factor (i.e. step length (right leg) at 1 km/h), as well as combinations of the most predictive factors (B-D) for falls.

*CoP* center of pressure, *COV* coefficient of variance, *med-lat* mediolateral.

### ROC curve analysis of principal determinants of fallers

**Figure 3:**
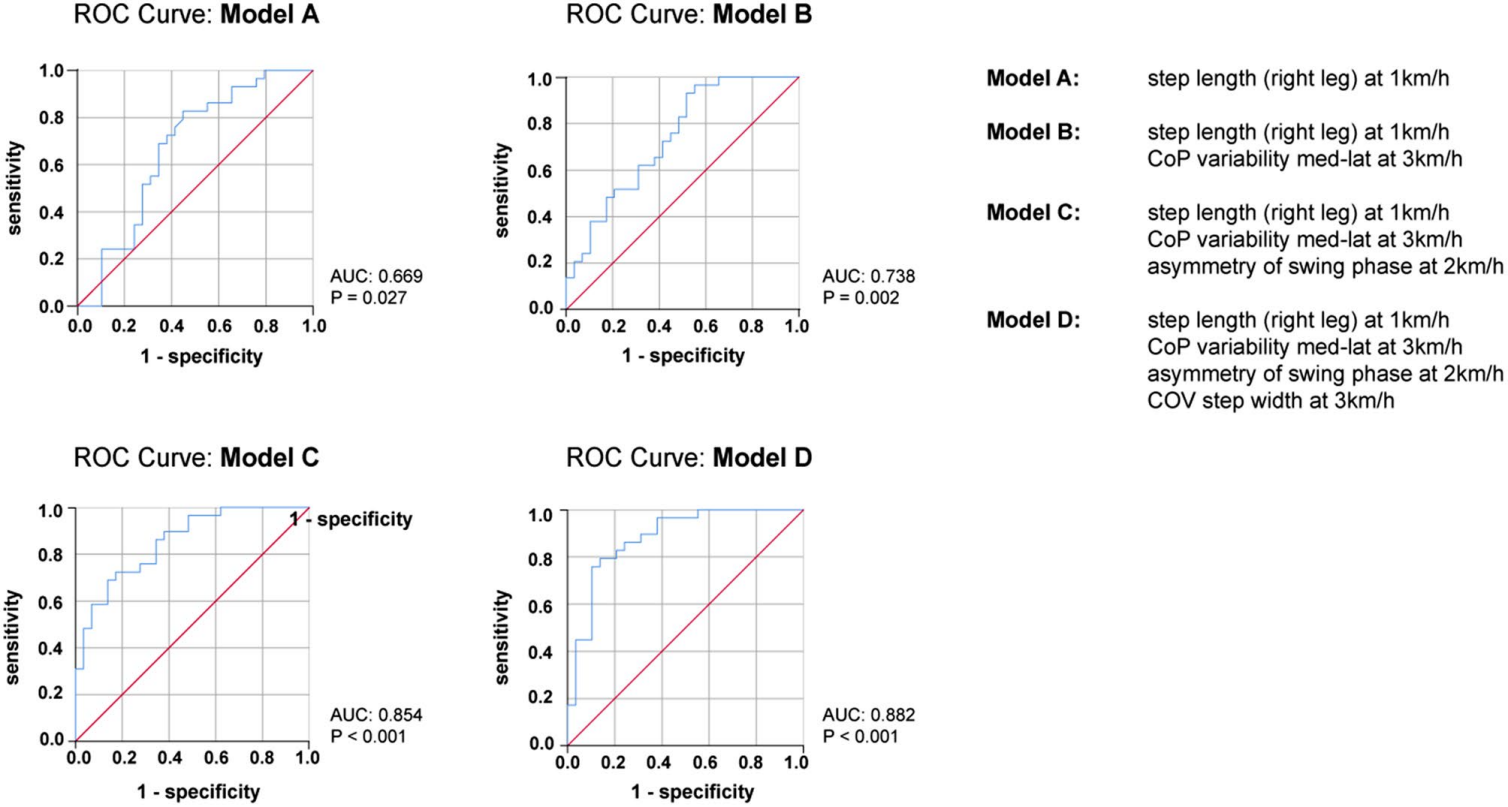
Receiver operator characteristics curves of the best predictors of falls. The best predictors of falls were further analyzed by receiver operator characteristics (ROC) curves to investigate the specificity and sensitivity of these variables. ROC curves were assessed for the best single predictor (Model A; top left), as well as for the best combinations of predictors (Model B–D). AUC and P-values of the ROC curves are highlighted at the right bottom corner of each panel. *AUC* area under the curve, *CoP* center of pressure, *COV* coefficient of variance.

Detailed sensitivity and specificity characteristics of the strongest fall-related determinants (as defined by binary logistic regression analysis) were further assessed by receiver operator characteristics (ROC; Fig. 3). The best single parameter (i.e. right step length at 1 km/h) reached a ROC area under the curve (AUC) of 0.669 suggesting valid but rather modest ability to correctly classify fallers and non-fallers. The combination of different fall-related determinants reached AUC values of 0.738, 0.854 and 0.882 for combinations of 2, 3 and 4 variables, respectively (Fig. 3). These combinations thus reveal fair to good ability to distinguish between fallers and non-fallers.

### Fall-related functional characteristics in subjects with inflammatory CNS diseases

Analysis of functional determinants of falls in patients with inflammatory CNS diseases (i.e. multiple sclerosis (n = 37), clinically isolated syndrome (n = 2)) identified enhanced variability of step width at 3 km/h as only factor associated with falls. The power of this factor is considered moderate to low (accuracy: 61.5%; specificity: 77.3%; sensitivity: 41.2%; Nagelkerke coefficient of determination R^2^ = 0.151). ROC analysis revealed an AUC of 0.698 (p = 0.08). The covariates age (P = 0.828) and gender (P = 0.140) did not have an influence on the analysis.

## Discussion

This study aimed at determining the principal postural and gait impairments that are associated with falls in patients with various neurological disorders. A comprehensive set of kinetic gait parameters, standardized clinical walking tests and posturographic parameters was assessed to identify the main determinants of falls in our cohort of neurological patients. The strongest fall-related functional impairment was a reduction of right step length at slow walking speed. The gait phenotype that was most strongly associated with falls was characterized by reduced step length, restricted mediolateral CoP variability, enhanced swing phase asymmetry and increased variability of step width. Our results highlight the main biomechanical characteristics of fallers with neurological disorders. The identified parameters represent paramount markers of enhanced fall risk in various neurological gait disorders that should be considered in physiotherapeutical strategies that aim at preventing falls in neurological patients. Moreover, objective monitoring of these fall-related gait parameters might allow to optimize treatments for gait disorders (e.g. medications, physical therapy etc.) in the clinical setting.

The strongest spatio-temporal gait determinants of falls identified in this study (i.e. reduced step length) were previously described as predictors of falls in healthy elderly people^4,6,12,13^ . However, in all of the mentioned studies fallers and non-fallers walked at their self-selected speeds, often resulting in slower walking speeds in fallers than non-fallers. As most gait parameters (including step length) show significant adaptations in response to changing gait speeds^37,38^, differential walking speeds between the investigated groups confound an accurate analysis of group characteristics. In the present study, we assessed kinetic walking parameters at three different, fixed walking speeds in all patients. This allowed us to perform precise inter-subject comparisons that are not confounded by varying gait speeds between participants. Analogous to our study, Barak et al.^2^ investigated stride length at fixed gait speeds and found shorter stride lengths in elderly fallers vs. non-fallers. Whereas there is robust evidence for step length reduction being a valid biomechanical determinant of falls in healthy elderly subjects, there is only sparse data on neurological patients. A recent study reported a significant reduction in step length in fallers vs. non-fallers in chronic stroke survivors^27^. Socie & Sosnoff^39^ found a non-significant tendency towards shorter steps in people with multiple sclerosis (MS) and previous fall history. Step length reduction might reflect a compensatory strategy of neurological patients to counteract impairments in muscle strength and balance function^40,41^. Whether step length reduction is a primary fall-related gait characteristic or a secondary adaptation in response to another gait impairment remains to be clarified. The right-sided shortening of step length in our study might be explained by the fact that most patients show asymmetric gait deficits which—in our population—lead to an overall step length reduction that is more pronounced on the right side.

Reduced variability of the CoP in the mediolateral dimension was identified as important determinant of falls in our cohort. Whereas other parameters of gait variability are usually enhanced in fallers vs. non-fallers in healthy elderly fallers^2,3,5–10,42^, mediolateral variability of the COP was reduced in fallers vs. non fallers in our study. This finding conforms to a previous study that investigated mediolateral gait dynamics during walking in stroke survivors: the authors reported reduced mediolateral pelvis displacement in fallers vs. non-fallers with chronic stroke^43^. Reduced mediolateral variability might be an adaptation to stabilize the gait pattern which is also found in fall-prone populations other than stroke patients.

Asymmetric walking patterns are frequently observed in patients with neurological disorders and usually derive from unilaterally accentuated weakness, spasticity or sensory deficits^44^. We found that left–right asymmetry of swing phase is a biomechanical hallmark of fallers in patients with neurological disorders. Swing phase asymmetry has been reported previously in people with MS^37,45,46^ and other studies reported enhanced gait asymmetry in people with Parkinson’s disease and stroke^47–49^. Kasser et al.^45^ conducted a logistic regression analysis to assess different balance, gait and strength parameters that are associated with falls in a group of female patients with MS. The authors identified stance time asymmetry as important determinant of falls in this population. Enhanced left–right asymmetry of gait parameters often results in claudication and reduced dynamic stability, thus likely increasing patients' fall risk.

Our results demonstrated increased step width variability in fallers vs. non-fallers. This is in agreement with previous studies on fall-related determinants in healthy elderly subjects^3,7,12^ . Additionally, Rochester et al.^50^ reported increased step width variability in fallers with Parkinson’s disease in comparison to healthy controls. The aforementioned studies underpin the value of this gait parameter to serve as biomechanical marker of gait instability and falls.

Enhanced variability of step width was identified as single fall-related determinant in a sub-analysis in patients with inflammatory CNS diseases (i.e. multiple sclerosis (MS; n = 37), clinically isolated syndrome (CIS; n = 2)). Whereas previous studies identified this gait parameter as important hallmark of MS-related gait deficits^37,51,52^ there are, to our knowledge, no findings that related enhanced step width variability with increased risk of falls in patients with MS or CIS.

Our data demonstrates accentuated gait pattern differences between fallers and non-fallers with increasing gait velocity. This indicates that faster walking speeds might be more sensitive to detect fall-related gait pathologies, which is in line with previous reports on elderly healthy subjects^2,6^ and patients with MS^53^. These findings highlight the importance to assess gait patterns at different speeds, also including non-comfortable, high speeds that challenge patients and facilitate the detection of subtle gait abnormalities^29^.

In this study, we assessed a comprehensive set of spatio-temporal gait parameters, standardized clinical walking tests and posturographic outcomes in a mixed population of neurological patients and examined the ability of these parameters to differentiate fallers from non-fallers. The superior power of spatio-temporal gait parameters to separate fallers from non-fallers might be explained by the fact that most falls actually occur during walking^54^. Enhanced fall prediction by spatio-temporal gait parameters vs. clinical gait tests is in line with earlier reports^6,10,14,27,55,56^ and might derive from enhanced sensitivity and objectivity of instrumented, technical outcome measures. Two studies that assessed computerized gait and balance measures, as well as clinical measures in patients with chronic stroke demonstrated that spatio-temporal gait parameters were better determinants of falls than posturographic and clinical parameters^43,49^. Summarized, these findings suggest that spatio-temporal gait parameters are a sensitive tool to assess fall-related functional impairments in neurological patients. The findings of our study do not allow to draw conclusion whether the identified functional determinants of falls are unique to neurological patients or whether they can also be found in other populations (e.g. elderly fallers).

Moreover, we used different fixed velocities 1, 2 and 3 km/h). A complete analysis of gait patterns across different walking speeds allows to assess different modalities of gait function (e.g. balance at slow walking; strength and coordination at higher speeds) in all subjects^29^, which valorizes the quality of our multimodal gait analysis.

Another strength is the comprehensive collection of gait and stance tests including instrumented kinetic gait analysis, clinical gait tests and posturographic stance analysis. Thus, we could show the primary eligibility of kinetic parameters to test for faller-specific gait adaptations.

Furthermore, for two reasons we see the heterogeneity of the sample as a strength to find faller-related adaptations of gait. First, a certain variety of neurological gait patterns (faller and non-faller) is needed to be able to detect a potential dominant parameter which contributes to a history of falls. Second, as even in patients with the same neurological disease gait pattern vary widely, we wanted to find a measurement technique which is sufficiently sensitive to that. To enhance the knowledge and treatment of neurological gait disorders this is an essential predisposition.

A limitation of this study is that the instrumented gait assessment on the treadmill might not allow to entirely transfer the findings to over ground walking. However, many studies showed that the basic locomotor pattern during treadmill walking is highly similar to walking over normal ground in subjects that were sufficiently familiarized to treadmill walking^34,57–60^. A strength of treadmill-based gait analysis is that gait speed can be fixed, thus allowing for an accurate inter-subject comparison of gait parameters without confounding effects of differential walking speed between subjects^37,38^. Moreover, the treadmill allows to sample numerous, continous step cycles that enhances the power of gait analysis. A limitation of this study is the exclusion of subjects that were not able to walk unassisted at 3 km/h on the treadmill likely caused a bias towards good walkers. The use of handrails, however, substantially influences subjects’ walking patterns, thus confounding an accurate biomechanical gait analysis. Furthermore, our population revealed a high amount of people with MS, whereas some of the other neurological diagnoses were rather rare. Hence, our data might primarily reflect findings of people with MS and do less apply to other neurological cohorts. Another limitation is that due to the retrospective design of the study data on cognitive function is not available. Subjects with major cognitive deficits were excluded. However, we cannot exclude that mild cognitive deficits have influenced the fill out of the questionnaire and the performance of the walking tests. Furthermore, fear of falling was not tested but could have influenced the walking pattern during all tests. As this factor cannot be eliminated it would be helpful to evaluate if the faller-specific adaptations are also correlated to the magnitude of fear of falling. Future studies should include the testing of cognitive function and fear of falling to see their influence on faller-specific gait adaptations. Another important point is the retrospective design of the fall questionnaire which might lead to an underestimation of fall incidents as the patient might not remember fall events in the long past. In order to reduce the risk of recall bias we restricted on the retrospective fall assessment to six months.

## Conclusion

Our findings highlight the principal functional impairments that are related to falls in a mixed population of neurological patients. The results suggest that multimodal gait analysis is superior to posturographic or clinical walking outcomes in differentiating fallers from non-fallers. Reduced step length at slow walking speed was the strongest single determinant of fallers, probably reflecting a compensation strategy for impaired muscle strength and balance function. Gait asymmetry and altered variability values of gait parameters were additional gait characteristics of fallers. Our findings emphasize the most prominent locomotor determinants of falls and thus present potential key targets for future interventions aiming at preventing falls in neurological patients.
